# A Green Stable Antifouling PEGylated PVDF Membrane Prepared by Vapor-Induced Phase Separation

**DOI:** 10.3390/membranes12121277

**Published:** 2022-12-16

**Authors:** Hana Nur Aini, Irish Maggay, Yung Chang, Antoine Venault

**Affiliations:** R&D Center for Membrane Technology, Chung Yuan Christian University, Taoyuan 32023, Taiwan

**Keywords:** green membrane, antifouling membrane, triethyl phosphate, VIPS process

## Abstract

While green solvents are being implemented in the fabrication of polyvinylidene fluoride (PVDF) membranes, most are not compatible with the vapor-induced phase separation (VIPS) process for which relatively low dissolution temperatures are required. Additionally, preparing antifouling green membranes in one step by blending the polymer with an antifouling material before inducing phase separation remains extremely challenging due to the solubility issues. Here, the green solvent triethyl phosphate (TEP) was used to solubilize both PVDF and a copolymer (synthesized from styrene monomer and poly(ethylene glycol) methyl ether methacrylate). VIPS was then used, yielding symmetric bi-continuous microfiltration membranes. For a 2 wt% copolymer content in the casting solution, the corresponding membrane P2 showed a homogeneous and dense surface distribution of the copolymer, resulting in a high hydration capacity (>900 mg/cm^3^) and effective resistance to biofouling during the adsorption tests using bovine serum albumin, *Escherichia coli* or whole blood, with a measured fouling reduction of 80%, 89% and 90%, respectively. Cyclic filtration tests using bacteria highlighted the competitive antifouling properties of the membranes with a flux recovery ratio after two water/bacterial solution cycles higher than 70%, a reversible flux decline ratio of about 62% and an irreversible flux decline ratio of 28%. Finally, these green antifouling membranes were shown to be stable despite several weeks of immersion in water.

## 1. Introduction

The extensive use of toxic solvents during membrane fabrication challenges the claim that membrane filtration is a green separation technology. Additionally, as more stringent regulations are being implemented in the utilization of solvents, membrane scientists have started to search for more suitable alternatives and shifted their focus towards membrane fabrication using green or greener solvents [1,2,3,4,5]. Among them, cyrene [6]; dimethyl isosorbide [2]; gamma-butyrolactone [7] and cyclic carbonate solvents (ethylene carbonate, propylene carbonate and butylene carbonate) [8] but also dimethyl sulfoxide (DMSO) [9,10] or triethyl phosphate (TEP) [11,12] have emerged as potential solutions to make a membrane fabrication green.

The vapor-induced phase separation (VIPS) process is an adequate process to prepare polyvinylidene fluoride (PVDF) membranes in the microfiltration range [13], with either rough spherulitic structures having a very large water contact angle (WCA), ideal for the gravity-driven separation of oil and water from oi-rich wastewater, or with bi-continuous structures, appropriate for the pretreatment of general wastewater. VIPS is known as being a more controllable process than the more traditional wet immersion process (or nonsolvent-induced phase separation (NIPS) process), because the nonsolvent/polymeric system is initially a vapor/liquid interface. VIPS membranes can also be seen as complement to wet immersion membranes that usually fall into the ultrafiltration domain. It somehow competes with temperature-induced phase separation (TIPS) membranes, as far as the pore size range is concerned. Nevertheless, TIPS membranes are often hollow fibers [11,14,15,16], while flat-sheet VIPS membranes are more commonly prepared [13]. The fabrication of VIPS membranes requires the utilization of solvents, and one underestimated challenge that then arises is the range of temperatures to obtain a polymeric solution. Some earlier studies on PVDF membranes revealed the relationship between the temperature of the dissolution, the morphological features of the membrane and the membrane properties [17,18,19]. Additionally, as the temperature in the VIPS chamber is relatively low (likely below 50 °C to make hand-casting doable), the polymer should remain soluble in the solvent at this temperature. A variety of solvents meets this requirement, but many are toxic. Nevertheless, DMSO and TEP, considered as green, are viable options. They both possess a small interaction distance with PVDF (as defined from the solubility parameters and are reported to be 1.08 for TEP and 4.54 for DMSO [20]), which facilitates the formation of a homogeneous casting solution even at relatively low temperatures. Dimethyl sulfoxide has been successfully employed to tailor the properties of PVDF membranes prepared by the VIPS process [21]. TEP was also reported in the preparation of PVDF membranes by both wet immersion [19,22] and VIPS [23]. However, there is a lack of investigation concerning the formation of green and antifouling membranes by VIPS. Their fabrication requires using an antifouling additive such as a hydrophilic polymer or an amphiphilic copolymer in a blend with the PVDF/solvent system, which logically complicates the task of obtaining a homogeneous casting solution at low temperature. DMSO has recently been reported as a solvent additive to form effective antifouling PVDF-based membranes in one step by wet immersion [24], but there is a gap concerning the preparation of MF antifouling green membranes in one step, i.e., from a polymer/additive/green solvent blend.

To answer this need, PVDF was blended in TEP together with a random copolymer synthesized from styrene monomer and poly(ethylene glycol) methyl ether methacrylate (PEGMA), referred to as P(S-*r*-EGMA). This copolymer was reported earlier as an effective material for the formation of antifouling membranes using traditional (yet toxic) solvents [25]. Then, MF flat-sheet membranes were prepared by the VIPS process. The objectives of this work were to fully characterize and rationalize the structures of these novel green membranes, as well as their surface chemistry and wetting properties, before moving onto the assessment of their antifouling properties. Symmetric membranes with a homogeneous surface distribution of the copolymer were obtained, able to efficiently trap water and mitigate biofouling by a larger variety of biofoulants (proteins, bacteria and blood cells) in static (adsorption tests) and dynamic (filtration tests) conditions.

## 2. Materials and Methods

### 2.1. Materials

Styrene was bought from Showa Co. PEGMA (Mn 500), and toluene and hexane were purchased from Sigma-Aldrich. Azobisisobutyronitrile (AIBN) was obtained from Alfa Aesar Co. (Haverhill, Massachusetts, United States) Poly(vinylidene difluoride) (PVDF Kynar^®^ HSV 900) with an average molecular weight of 1,500,000 g/mol was obtained from Arkema (Colombes, France). It was rinsed with successive baths of deionized (DI) water and methanol before use. Triethyl phosphate (TEP) was purchased from Tokyo Chemical Industry and used without any further purification. DI water was produced in our laboratory with a Purelab^®^ water purification system obtained from Elga-Veolia. *Escherichia coli* was purchased from the Hsinchu Bioresource Collection and Research Center (Hsinchu, Taiwan), while whole blood was obtained from a pool of healthy volunteers at the Taipei MacKay Memorial Hospital (New Taipei, Taiwan). Bovine serum albumin (BSA) was purchased from Sigma-Aldrich (St. Louis, MO, USA).

### 2.2. Methods

#### 2.2.1. Polymer Synthesis and Characterization

**Figure 1:**
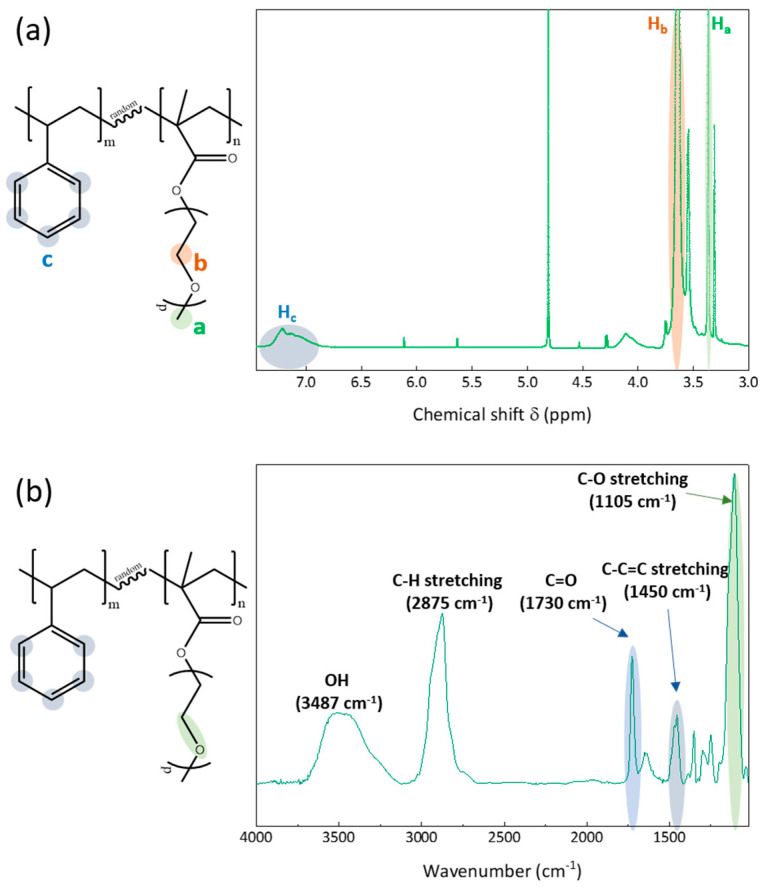
Characterization of the P(S-r-EGMA) copolymer by (**a**) 1H NMR and (**b**) FTIR spectroscopy.

The synthesis of the amphiphilic copolymer by free radical polymerization has been presented elsewhere [25] and is briefly reminded here. Styrene monomer, PEGMA oligomer and AIBN were mixed in toluene with a total solid content of 30 wt% and a monomer/initiator molar ratio of 350. These conditions were chosen based on our previous study that aimed at optimizing the copolymer composition for both the fouling mitigation of VIPS membranes and stability of the system [25]. After 30 min, the system was purged with nitrogen, and the temperature increased to 80 °C. After 24 h, the reaction was stopped by immersing the reaction flask in an ice bath. Finally, the copolymer was precipitated using hexane, then vacuum-dried, freeze-dried and stored at 4 °C before characterization and use in membrane preparation. As the copolymer has been presented before, its characteristic ^1^H NMR and FTIR spectra are briefly presented in this section as well (Figure 1). ^1^H NMR was obtained with a Bruker 600 MHz instrument using D-methanol solvent and permitted to determine the actual composition (39 mol% of polystyrene/61 mol% of PEGMA) of the copolymer from the peaks centered at δ = 7.1 ppm (5 protons labeled H_c_ of the aromatic group of styrene units) and at δ = 3.3 ppm (3 protons labeled H_a_ of the methoxy group of EGMA units). The FTIR analysis performed with a Jasco FT/IR-6700 spectrometer confirmed the presence of C–C=C (1450 cm^−1^, stretch [26]) brought by polystyrene and of both C=O (1730 cm^−1^, stretch [27]) and C–O (1350 cm^−1^, stretch [28]) of PEGMA in the copolymer. Finally, a gel permeation chromatography analysis was conducted with a Viscotek instrument using a OHpak SB-803 HQ column and DI water as the eluent to determine the molecular weight of the copolymer, found to be 133 kDa.

#### 2.2.2. Casting Solutions and Membranes Preparation

Casting solutions were prepared by dissolving P(S-*r*-EGMA) copolymer and PVDF in TEP under constant stirring. The copolymer content varied between 0 and 2 wt.%, while the PVDF content was fixed at 12 wt.%. The dissolution temperature was fixed at 60 °C. Once the polymers dissolved and the solution was homogeneous, stirring was stopped to allow degassing.

The zero shear rate viscosity at 25 °C of the casting solutions was determined with a DHR-2 rheometer (TA instruments, New Taipei City, Taiwan) using parallel plate geometry with a diameter of 20 mm (Peltier plate steel). The polymer solution was placed in between the two plates and the gap size set to 100 µm. A 10 s pre-shear at a maximum shear rate of 200 s^−1^ was performed. To avoid solvent evaporation from the rim, the system was covered with a solvent trap casing.

Dynamic light scattering tests were conducted at 25 °C with a Delsa^TM^ Nano S particle analyzer (Beckman Coulter, Brea, CA, USA) after diluting the polymer/solvent or polymer/copolymer/solvent solutions 10 times using TEP.

The membranes were prepared by the VIPS process in a chamber wherein the relative humidity and the temperature were fixed to 70% and 30 °C, respectively. At this temperature, the systems remained homogeneous (no visible phase separation nor gelation occurred). The solutions were hand-cast on glass substrates using a metallic casting knife with a clearance of 300 µm. After 20 min of exposure time to water vapors, the films were immersed in a bath of DI water, dried under atmospheric conditions and then stored in a refrigerator until use.

In this work, the membranes were labeled P0, P0.5, P1, P1.5 and P2, where P represents the P(S-*r*-EGMA) copolymer and the following number its content in the solution from which the membranes were prepared. Therefore, P0 represents the control virgin membrane (no copolymer).

#### 2.2.3. Pseudo-Ternary Phase Diagram Determination

PVDF/TEP/water ternary and PVDF/P(S-*r*-EGMA)/TEP/water pseudo-ternary phase diagrams were determined by the cloud point method. Briefly, this method consists of preparing a set of polymeric solutions with varying PVDF contents and then adding water dropwise under constant stirring. Once the system becomes permanently cloudy, the cloud point has been reached, and the corresponding composition can be reported in the diagram. The set of obtained compositions at the cloud point is an experimental evaluation of the binodal line. In the case of the pseudo-ternary phase diagram, the PVDF to P(S-*r*-EGMA) weight ratio was fixed at 6:1 (corresponding to the composition of the casting solution used to prepare the P2 membrane). All phase diagrams were determined at 60 °C, similar to the temperature at which the casting solutions were prepared.

#### 2.2.4. Membranes Characterization Tests

The structure of the membranes was observed by SEM using a Hitachi 4800 instrument (Tokyo, Japan). Samples were sputter-coated with gold before observation. The membrane pore size was assessed with a capillary flow porometer (PMI). The membrane porosity was determined by gravimetric measurements using butanol. The formula used for the porosity has been reported elsewhere [25].

The surface chemistry of membranes was analyzed by FTIR and by mapping FTIR, using Jasco equipment (FTIR-6700 unit and IRT-5200 microscope, Tokyo, Japan). The resolution of each analysis was 4 cm^−1^, and 32 scans/spectrum were acquired. For mapping tests, the aperture was 30 µm.

The water contact angle (WCA) in the air was determined with an optical contact angle system (OCA 15 EC, DataPhysics Instruments, Charlotte, USA). The hydration capacity was measured on 1.3 cm diameter disk membrane samples. For this, the membranes were dried, weighed and then immersed in DI water for 24 h. Afterwards, they were weighed again and the difference between the wet and dry weights per unit surface area taken as the hydration capacity of the sample. For the WCA and hydration capacity measurements, 5 independent tests were carried out.

#### 2.2.5. Antifouling Tests in Static Condition

The antifouling properties of the membranes in the static condition were tested using *Escherichia coli* bacteria, whole blood and BSA. In all cases, 1.3 cm diameter membrane samples were incubated with the biofouling solution at 37 °C after washing with a PBS solution, and 5 independent tests were performed.

Regarding the bacterial attachment tests, the samples were incubated for 24 h with 1 mL of *Escherichia coli* solution prepared according to a procedure earlier reported [25], and the bacterial solution was changed every 6 h. For the whole blood tests, the membranes were incubated with 1 mL of whole blood for 1 h. For the BSA adsorption tests, the samples were incubated with 1 mL of the protein solution (1 mg/mL in PBS). After the tests, the membranes incubated with whole blood were fixed using a 2.5% solution of glutaraldehyde for 4 h. The samples incubated with bacteria did not require any dyeing step due to the presence of a green fluorescent protein. For both the bacterial and blood adhesion tests, the membranes were observed with a confocal microscope (Nikon CLSM A1R instrument, Tokyo, Japan) and the images analyzed with ImageJ^®^ software (an open source software developed by the National Institutes of Health, USA). In the case of the BSA adsorption tests, the amount of adsorbed protein was evaluated by running a UV–Vis spectrophotometry analysis at 280 nm on the incubation solutions.

#### 2.2.6. Cyclic Filtration Tests

DI water/*Escherichia coli* solution cycles were performed to evaluate the antifouling properties of the MF membranes during filtration. Firstly, 5 cm disk membranes were immersed in ethanol and placed in a dead-end filtration cell. Then, DI water was filtrated at a pressure of 1.5 bar. After 30 min, the pressure was decreased to 1 bar, the operating pressure used for the test. Once a steady state was reached, the permeate weight was monitored for 30 min. Then, the DI water tank was disconnected from the module and replaced by a tank containing the bacterial solution (of initial concentration 8.5 10^8^–1.2 10^9^ cells/mL) and permeate weight monitored for another 30 min. Afterwards, the membranes were cleaned by a backflush procedure at 1 bar for 30 min to remove the reversible fouling. Then, a second DI water/*Escherichia coli* solution cycle was conducted, followed by a second membrane cleansing and the final DI water permeability measured for the last 30 min of the test. In this study, the flux recovery ratio (FRR, %), the total flux decline ratio (DRt, %), the reversible flux decline ratio (DRr, %) and the irreversible flux decline ratio (DRir, %) could be determined from the initial water flux ($J_{w,0},\;L/m.h.bar$), the final water flux ($J_{w,f},\;L/m.h.bar$) and the final bacterial solution flux ($J_{EC,f},\;L/m.h.bar$) as follows:

(1) $$FRR=\frac{J_{w,f}}{J_{w,0}}\times 100$$

(2) $$DR_{r}=\frac{J_{w,f}-J_{EC,f}}{J_{w,0}}\times 100$$

(3) $$DR_{ir}=\frac{J_{w,0}-J_{w,f}}{J_{w,0}}\times 100$$

(4) $$DR_{t}=DR_{r}+DR_{ir}$$

#### 2.2.7. Stability Tests

The stability of the membranes was evaluated by immersing the membranes for several weeks in DI water. Then, mapping FTIR (same instrument as mentioned in Section 2.2.4) was utilized to visualize the effect of the immersion on the surface distribution of the copolymer. Maps were acquired at 1730 cm^−1^ on samples of 1.5 mm × 1.5 mm and with an aperture of 30 µm. Additionally, the relative amount of remaining copolymer could be tracked by measuring the surface area ratio of the peak at 715–789 cm^−1^ or 1370–1450 cm^−1^ (PVDF) to the peak at 1720–1740 cm^−1^ (P(S-*r*-EGMA) copolymer).

## 3. Results and Discussion

### 3.1. Physical Characterization of the PEGylated Membranes and Aspects of Membrane Formation

**Figure 2:**
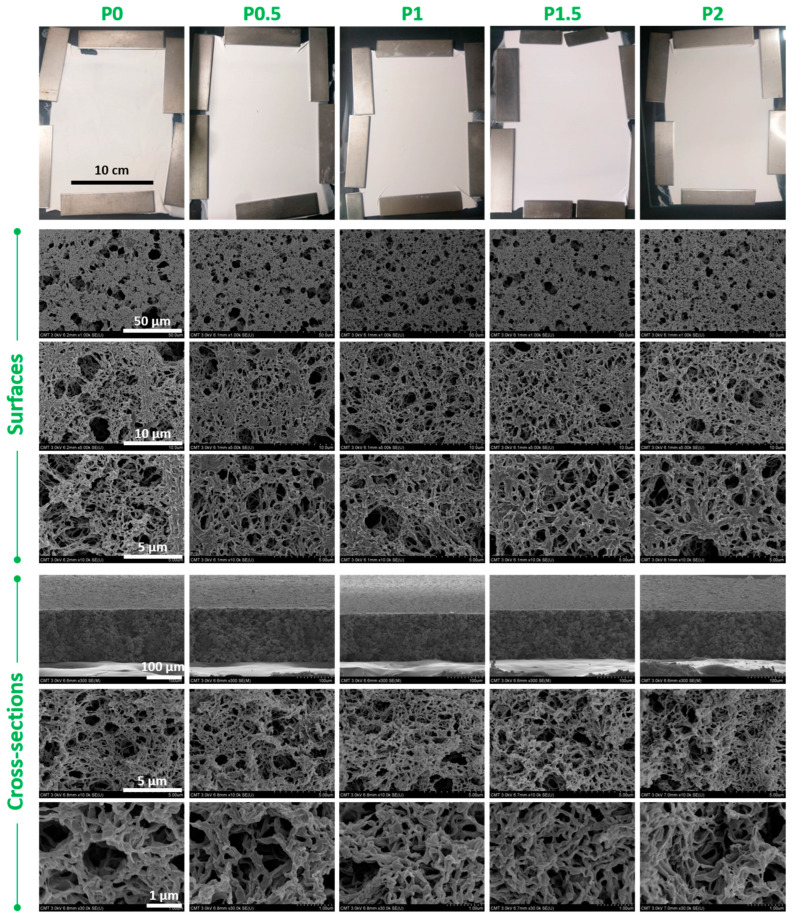
Photographs and scanning electron microscope of the virgin (P0) and PEGylated (P0.5 to P2) VIPS membranes. On each raw, the scale bar is similar.

**Figure 3:**
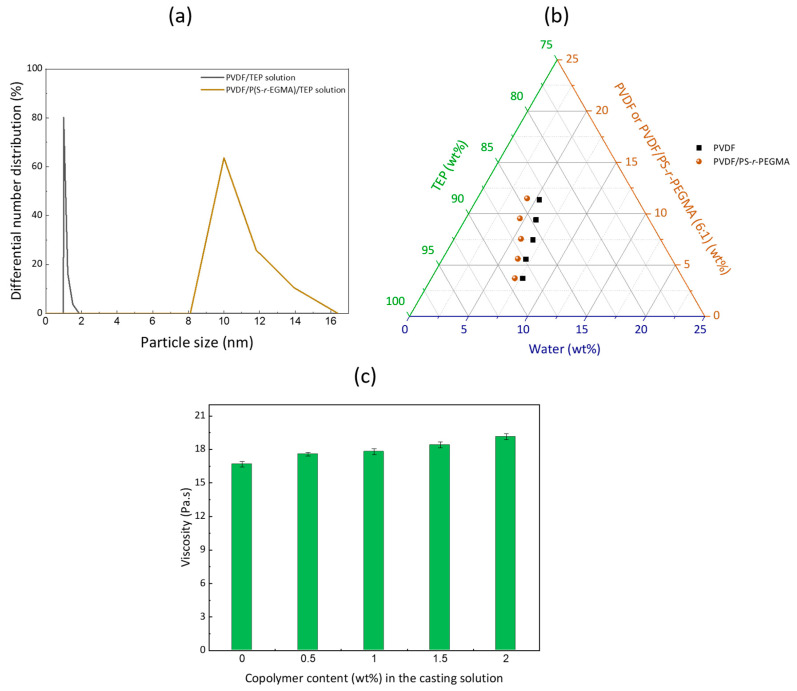
Influence of the copolymer on (**a**) particle size distribution in the casting solution, (**b**) the position of the cloud point curve associated with the thermodynamic stability of the polymer/solvent system when exposed to a nonsolvent at 60 °C and (**c**) the zero shear rate viscosity of the casting solutions at 25 °C (all solutions contained a fixed (12 wt%) amount of PVDF).

In this study, large membranes could be hand-cast. The resulting films visually seemed homogeneous, whitish and matte (Figure 2). These visual observations suggest that the membranes formed are highly porous, as light is absorbed by the numerous pores decorating the surface. This is reasonable, as VIPS applied to solutions containing PVDF commonly leads to highly porous membranes in the MF domain. Then, the SEM images permit to define in detail the type of structure obtained. Symmetric bi-continuous structures with large pores were obtained, implying that the membranes were formed by L/L phase separation rather than by crystallization. The former occurs if exchanges of a solvent and nonsolvent are fast, while crystallization is known to dominate when exchanges are slow [29]. The addition of a copolymer did lead to an increase in particle size in the system, but it remained small enough (well below the order of submicron) to not induce any observable precipitation (Figure 3a). P(S-*r*-EGMA) destabilizes the system—that is, switches the cloud point curve towards the polymer–solvent axis (Figure 3b). This addition should promote the formation of structures characteristic from L/L phase separation (either cellular-like or bi-continuous), because the binodal is reached upon the smaller addition of a nonsolvent compared to without a copolymer. It is reasonable to assume that the smaller amount of nonsolvent needed to induce phase separation (i.e., a smaller-area demixing gap on the ternary diagram) correlates to faster phase separation. Additionally, the introduction of a copolymer increased the zero shear rate viscosity in a small extent only (from 16.7 ± 0.5 Pa.s for P0 to 19.2 ± 0.3 Pa.s for P2.0, Figure 3c) because a large molecular weight PVDF was used and only small amounts of copolymer added. It is believed that this change was too small to have significantly influenced the solvent/nonsolvent exchange rates. Therefore, the change in viscosity did not play a major role on the membrane structure, which could explain why similar morphologies were obtained with (P0.5 to P2 membranes) or without (P0 membrane) copolymers. The fact that bi-continuous structures were obtained in all cases would even suggest that membrane formation occurred by spinodal decomposition (SD) rather than by nucleation and growth (NG) and that there was no coarsening of the domains after phase inversion [30]. Su et al., studied the transition between SD and NG in nonsolvent-induced phase separation, and their results suggested that casting solutions with a higher tendency to gel would lead to bi-continuous membranes [31]. Several criteria are favorable to solution gelation (hence, to SD), including a high molecular weight for the polymer, a low solvency of the solvent or the presence of additives [29]. Here, two out of three criteria are met, since the polymer used has a high molecular weight PVDF (1,500,000 g/mol) and, as a copolymer (PS-*r*-PEGMA), was added to the systems. Finally, the retainment of the bi-continuous structure after phase inversion by SD is facilitated, because all solutions can readily gel (again, mostly due to the large molecular weight of the polymer). It means that the structure cannot evolve (or hardly) after phase inversion, which would have led otherwise to the formation of cellular domains after coalescence of the polymer chains.

**Figure 4:**
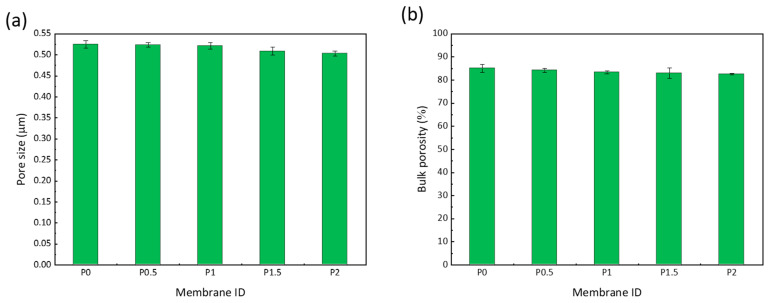
Influence of the copolymer on (**a**) the membrane pore size and on (**b**) the membrane bulk porosity.

Finally, the pore size and porosity were also determined and the results shown in Figure 4. For the unmodified membrane (P0), the pore size and porosity were found to be 0.52 µm and 85.1%, respectively, while, for the membrane prepared from a casting solution containing 2 wt% copolymer (P2), the pore size and porosity were 0.5 µm and 82.6%, respectively. The large pore size and porosity are again characteristic of the type of process used to fabricate the membrane. The slightly lower pore size and porosity for P2 compared to P0 are reasoned by the higher volume fraction occupied by the polymeric chains in P2 containing both PVDF (12 wt%) and PS-*r*-PEGMA (2 wt%). As seen earlier from the SEM images of Figure 2 showing the total cross-sections, the membranes all have a similar thickness. Therefore, the membrane volume of P0 and P2 are similar, which implies that the volume fraction occupied by the polymeric chains in P2 is slightly larger or that the volume fraction occupied by the pores is lower, giving rise to a somewhat smaller pore size and bulk porosity.

### 3.2. Characterization of the Surface Chemistry of the PEGylated Membranes

**Figure 5:**
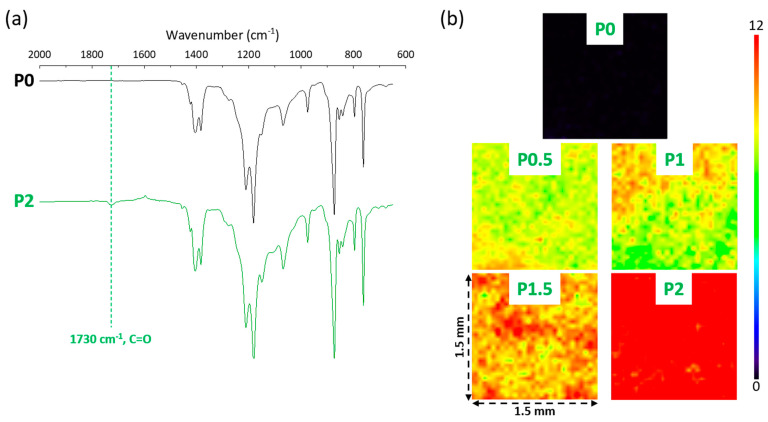
FTIR characterization of the PEGylated membranes. (**a**) ATR FTIR spectra of the P0 and P2 samples. (**b**) Mapping FTIR at 1730 cm^−1^ on 1.5 × 1.5 mm samples, realized with an aperture of 30 µm.

Membrane surfaces were analyzed by FTIR to evidence the presence of the PEGylated copolymer. In Section 3.1, an intense signal could be detected at wavenumber 1730 cm^−1^ corresponding to the C=O stretch of the PEGMA moieties. It could also be identified on the ATR-FTIR spectra of the membranes shown in Figure 5a, although the signal intensity was not as intense, because PVDF dominates the membrane’s composition. Additionally, mapping of the surfaces was realized at 1730 cm^−1^ (Figure 5b). The results of this analysis are color-coded between dark blue (0, no functional group) and deep red (12, high density of the tracked functional group). They permit not only detecting qualitatively the presence of the functional group (hence, here, of the copolymer) but also assessing the surface chemical homogeneity of the membranes on quite large surface areas. These maps indicate that, as the concentration of copolymer in the casting solution increased from 0 to 2 wt%, more copolymers could be logically detected on the membrane surface (dominating colors switching from dark blue for P0, green-yellow for P0.5, green-yellow-orange P1, orange-red for P1.5 and red for P2). They also showed that one color dominates the map of P2, which suggests that this membrane is homogeneous, as far as its surface chemical composition is concerned.

### 3.3. Hydrophilic Properties of the PEGyaled Membranes

**Figure 6:**
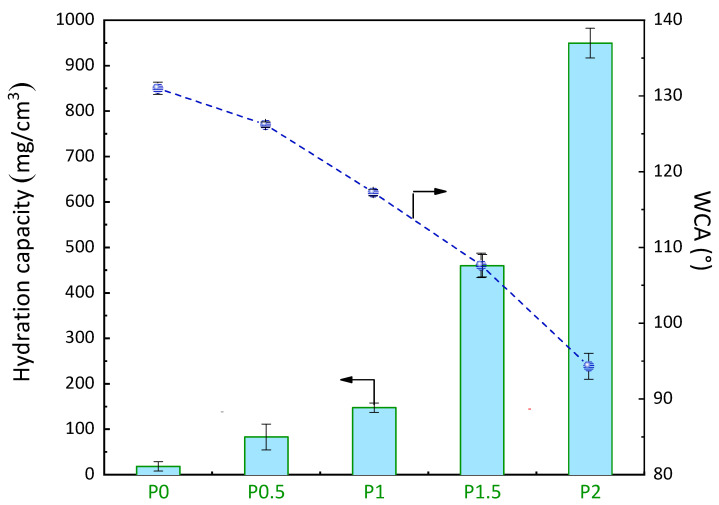
Effect of the PEGylated copolymer on the WCA and hydration capacity of the membranes.

It is well established that making an interface more hydrophilic is an efficient way to reduce biofouling [32,33]. Thus, measuring its surface and bulk hydration can provide intelligence on the ability of a membrane to mitigate fouling. Here, the WCA in the air and the hydration capacity were determined (Figure 6). The WCA of the virgin membrane (P0) was measured at 131° ± 1°. The high hydrophobicity of this membrane is attributed to both the intrinsic low surface free energy of the material (PVDF) [34] and to the highly porous nature of the membranes associated with the VIPS process. The latter significantly decreases its instantaneous wettability by water, as multiple surface pores trap air. In comparison, a PVDF membrane prepared by wet immersion would have a significantly lower WCA (<90° in our experience), as it would lead to denser and smoother surfaces. As a result of using VIPS, the modified membrane still showed a high WCA in the air (94° ± 2°), but it is undeniable that the modification could improve the surface wettability. For antifouling porous membranes, and, in particular, for those prepared by in situ modification, it is likely more appropriate to determine the capacity of the matrix to trap water in its bulk, as the amphiphilic material is distributed over the entire cross-section (although surface segregation [35] arises in a concentration gradient). Additionally, water trapping tests require immersing the membrane in an aqueous medium and fully wet it as it would be in a practical operation. Here, we determined the hydration capacity (HC) of the membranes by gravimetric measurements and could notice a drastic change in water trapping with the copolymer content in the membrane. While the virgin membrane floats, the PEGylated membranes could sink in the aqueous bath and then trap a large amount of water, as the highest HC measured with the P2 membrane was over 940 mg/cm^3^. This signifies that water can readily penetrate the PEGylated membrane and hydrate the polymer chains, which is an essential criterion for non-fouling.

### 3.4. Resistance to Escherichia coli Attachment

**Figure 7:**
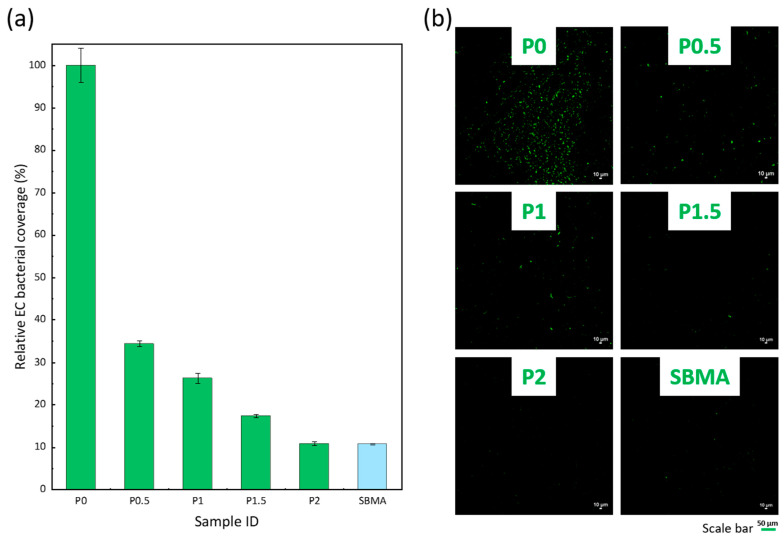
Effect of the PEGylated copolymer on the resistance to the attachment of *Escherichia coli*. (**a**) Relative bacterial attachment obtained by an image analysis. Results presented as mean ± SD (n = 9); (**b**) Representative confocal images for each condition. Control is a hydrogel of sulfobetaine methacrylate (SBMA).

The VIPS process permits to prepare PVDF membranes whose pore sizes fall into the MF range. Therefore, they can be helpful in pretreatment units for other pressure-driven membrane processes applied in wastewater treatment, seawater desalination, biomedical application, etc. to remove large biofoulants. Bacteria are considered as large biofoulants and are very common in wastewater [36,37,38]. Their pathogenicity depends on a number of factors (namely, infectivity and host resistance), but to avoid triggering disease, preventive measures such as the rejection of bacteria during the membrane treatment process should be implemented. MF membranes can be suitable to reject a large proportion of bacteria, but this will then result in bacterial attachment on the membrane surface, which can be reversible or irreversible, depending on the surface properties. To simulate attachment, membranes were incubated in a bacterial solution for 24 h and then observed by confocal microscopy. The results displayed in Figure 7 prove the efficiency of the modification. As little as 0.5 wt% copolymer in the casting solution permits reducing the bacterial attachment by 65%. Then, it could be further decreased to reach the minimum attachment using the P2 membrane (11% relative attachment or 89% decrease compared to the P0 membrane). Surface modifications with PEG, PEGMA or derivatives of ethylene glycol have been proven to be an effective way to inhibit bacterial adhesion on model or dense surfaces [39,40,41,42]. Here, it is shown that PEGMA moieties in the P(S-*r*-EGMA) copolymer are also effective in reducing biofouling in porous membranes despite their propensity to promote the physical attachment of bacteria on surface pores, providing a homogeneous and dense distribution of the antifouling units on the surface (as shown in Figure 5b). The lowest attachment measured on the P2 membrane is comparable to that measured on a zwitterionic hydrogel used as the control and made of sulfobetaine methacrylate (SBMA), which proves again the efficiency of the modification.

### 3.5. Resistance to Blood Cells Attachment

**Figure 8:**
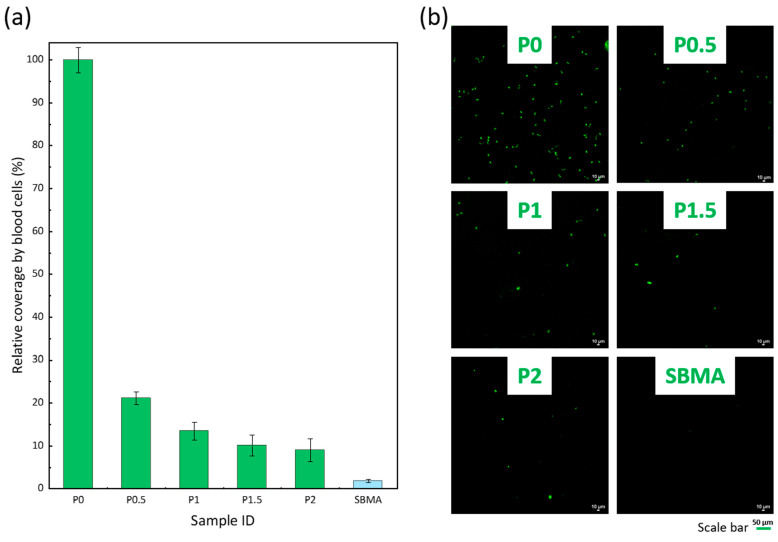
Effect of the PEGylated copolymer on the resistance to the attachment of cells from whole blood. (**a**) Relative surface coverage by blood cells obtained by an image analysis. Results presented as mean ± SD (n = 9). (**b**) Representative confocal images for each condition. Control is a hydrogel of sulfobetaine methacrylate (SBMA).

As mentioned earlier, the structures of the prepared membranes make them suitable for rejecting large biofoulants. Blood cells also fall into this category, and MF membranes can be used in biomedical applications in contact with blood, such as, for example, in leukodepletion [43]. Interactions of blood cells with the membrane material can quickly lead to fouling. To evaluate the efficacy of the membranes in resisting this type of biofouling, the membranes were incubated with whole blood and observed by confocal microscopy. The results presented in Figure 8 reveal numerous cells adhering on the P0 sample. In a previous study, Chang et al., emphasized the lack of blood compatibility of PVDF membranes and showed that full-scale platelet adhesion occurred after contact of the membranes with platelet-rich plasma [44]. More recently, An et al., grafted polyacryloylmorpholine with the aim of improving the hemocompatibility of PVDF membranes used in hemodialysis and showed the beneficial effect of the amphiphilic side chains on the reduction of protein adsorption and on the decrease of the hemolysis rate [45]. Here, the P(S-*r*-EGMA) copolymer plays a similar role and significantly contributes to mitigating blood cell attachment on the membrane, and a 90% reduction in cell adhesion was measured with the P2 sample. It implies that the as-prepared green membranes improved the hemocompatibility compared to the unmodified membrane, which would be essential for application in biomedical devices.

### 3.6. Resistance to Protein Adsorption

**Figure 9:**
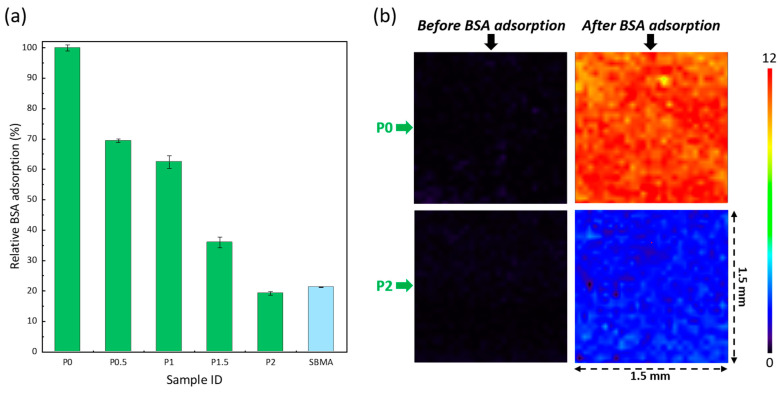
Effect of the PEGylated copolymer on the resistance to the adsorption of bovine serum albumin. (**a**) Relative surface coverage as determined by an analysis by UV–Vis spectroscopy at 280 nm. Results presented as mean ± SD (n = 3); 100% corresponds to 82 µg/cm^2^. Control is a hydrogel of sulfobetaine methacrylate (SBMA). (**b**) Mapping FTIR at 1650 cm^−1^ on 1.5 × 1.5 mm samples, realized with an aperture of 30 µm.

Proteins foul membranes at a lower scale than cells, as their small size facilitates their entrapment in the porous structure. BSA is commonly employed as a model protein to study the fouling of porous membranes in adsorption tests [28,46,47] or during cyclic water/protein solution filtration [48,49,50]. Here, adsorption tests were conducted rather than filtration tests (cyclic filtration were conducted with another type of biofoulant as MF membranes were prepared). The membranes were incubated with a 1 mg/mL BSA solution and adsorption analyzed by UV–Vis spectroscopy for quantification of the antifouling property (Figure 9a). Additionally, FTIR maps (Figure 9b) were acquired at 1650 cm^−1^ (amide group of BSA [51]) to assess the protein distribution on the membrane surface. The results indicate that protein adsorption could be reduced by 80% (corresponding to about 11 µg/cm^2^ vs. 82 µg/cm^2^ for the virgin membrane). Compared to the results of previous sections in which larger biofoulants were used, it is undeniable that more fouling occurred. Nevertheless, it remained low and comparable with the adsorption level measured by Chiag et al. [52]. Interestingly, fouling on P2 membrane is homogeneous, since only one dominating color (light blue) composes the map. To some extent, it indicates that the surface distribution of the copolymer is homogeneous and corroborates the results of Figure 5.

### 3.7. Performances of the Membranes during Cyclic Water/Bacterial Solution Filtration

In order to demonstrate the reduced fouling propensity on the green membranes during operation, cyclic water/bacterial solution filtration tests were conducted. Bacteria were chosen, as these are large biofoulants, and VIPS membranes would normally be used to reject particles or microorganisms of similar size. Additionally, *Escherichia. coli* was used as the model, as it is relatively safe to handle.

Although the prime purpose of the test was to study biofouling during operation and not to develop membranes for the removal of bacteria from wastewater, we still determined the rejection. It was 86 ± 3% for the virgin membrane and 97 ± 2% for P2. These rejections may seem low considering the membrane pore size and that of the bacteria (rod-shaped bacteria of a length of about 2 µm and diameter 0.5 µm). However, it is known that the cell wall of Gram-negative bacteria gives them flexibility. As such, they can deform and squeeze through smaller pores than their size under a pressure gradient [53], preventing a100% rejection. The higher rejection measured for P2 compared to P0 is likely due to the antifouling effect of the copolymer. Fewer bacteria adhered on the surface of the P2 membrane and, so, were likely to be deformed and pass through the membrane under the action of the hydraulic pressure.

**Figure 10:**
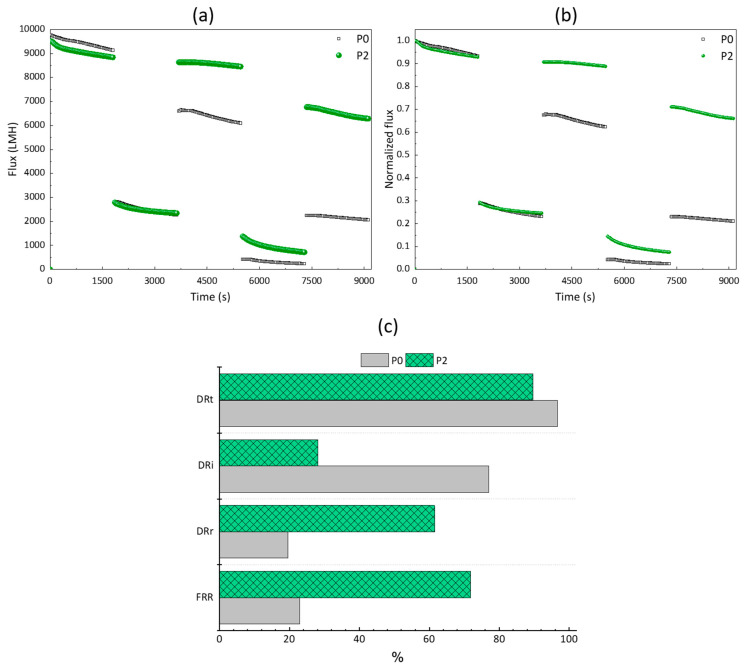
Effect of the PEGylated copolymer on the resistance to biofouling during filtration. (**a**) Permeability of the membranes vs. time during cyclic water/bacteria filtration. (**b**) Normalized flux (using initial permeability). (**c**) Flux indicator ratios. Filtrations were repeated 3 times, with a maximum standard deviation SD on the measurement as ±5%.

The permeate flux was recorded and is shown in Figure 10a. As membranes have slightly different permeability, data were also normalized using the initial flux to better visualize the effect of the copolymer on flux recovery (Figure 10b). Moreover, the important indicators associated with membrane fouling are displayed in Figure 10c. The membranes both exhibited a water permeability in the same range (about 9500 LMH at 1 bar), because their pore sizes were in the same range, and because they were both prewetted with alcohol prior to the tests. The effect of the copolymer is clearly seen at the end of the test, as the water permeability of P2 remained about 70% that measured initially. Contrariwise, that of P0 fell to less than 25% of its initial value. Irreversible fouling by the microorganisms occurred at the surface of P0 in a greater extent than at the surface of P2, as bacteria can readily interact with unmodified PVDF material even in static adsorption tests (Figure 7), as also recently reported by Ni et al. [54] or Maggay et al. [25]. Dynamic tests more severely foul the membrane as a result of the pressure gradient forcing the bacteria to interact with the surface and within the pores. The amphiphilic copolymer importantly reduced biofouling by the bacteria during filtration, and most of it was reversible (DRr of about 62% for P2), while irreversible fouling accounted for the majority of the total fouling in the case of the virgin membrane (DRi of about 77% for P0). Therefore, these membranes may be suitable for use in a membrane bioreactor or for microalgae harvesting, two applications wherein the membranes are in contact with large biofoulants (bacteria or microalgae) at high concentrations.

### 3.8. Stability of the Modification

**Figure 11:**
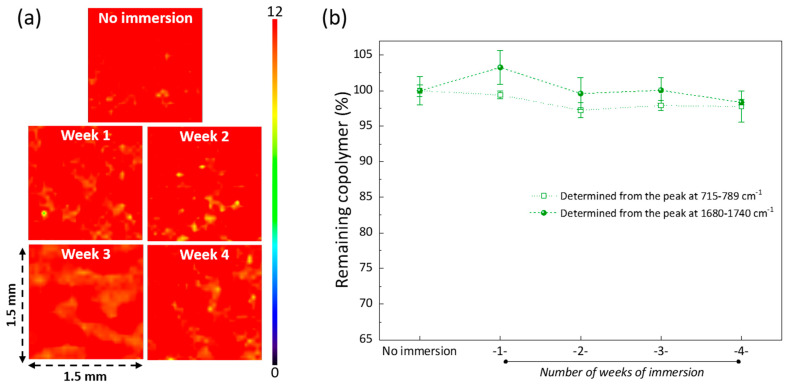
Stability of the modification. (**a**) Mapping FTIR images obtained at 1730 cm^−1^ (C=O of the PEGMA units in the copolymer) on 1.5 × 1.5 mm samples, realized with an aperture of 30 µm. (**b**) Quantitative analysis determined from the area ratio of a characteristic peak of PVDF (at 715–789 cm^−1^ or at 1370–1450 cm^−1^) to that of PEGMA (1720–1740 cm^−1^).

One major and commonly reported drawback of hydrophilic modifiers for membranes is that they can leach out of membranes, due to poor stabilizing interactions with the hydrophobic membrane material [55,56,57,58]. Amphiphilic copolymers such as the one employed in this study contain hydrophobic groups in which their main function is to provide stability to the system and reduce the propensity for leaching. To assess stability, membranes were continuously immersed in water, and their surface chemistry analyzed by FTIR. Maps tracking the presence of the C=O group of PEGMA were obtained (Figure 11a), while a quantitative analysis of the remaining copolymer was conducted (Figure 11b). The results clearly indicated that very little leaching occurred, with 98–100% copolymer remaining in the membrane (depending upon the peak of PVDF used for the analysis). Therefore, it can be concluded that the modification is stable. Lin et al., reported that commercial PVDF membranes coated with block copolymers of similar chemical composition could be used in a membrane bioreactor for several weeks without apparent decreasing in the membrane performances (other than an increase in the transmembrane pressure attributed to reversible fouling that could then be addressed by membrane washing) [59]. Thus, the stability of a similar polymer/amphiphilic copolymer has been observed. Here, the membranes were not coated but instead modified by physical blending. As such, the copolymer is entangled in the matrix polymer chains, which contributes to the high stability and makes the system potentially suitable for long-term operations.

## 4. Conclusions

This work presented the formation of antifouling and green microfiltration membranes by the VIPS process. TEP was employed as a green solvent to form homogeneous polymeric systems containing PVDF, the main membrane polymer, and P(S-*r*-EGMA) copolymer, an antifouling copolymer. Relatively low temperatures (60 °C) could be used to prepare the casting solutions, hence demonstrating that this green solvent is suitable for the formation of antifouling membranes in one step by VIPS or by other phase inversion processes that require low dissolution temperatures. The membranes obtained were bi-continuous, highly porous and with a homogeneous surface distribution of the copolymer. Their ability to trap a large amount of water resulted in the efficient mitigation of biofouling tested with various biofoulants (BSA, *Escherichia coli* and whole blood) in static and dynamic conditions. In addition, the membranes remained stable even after a long immersion in DI water (>98% copolymer remaining after 4 weeks). All in all, this work proves that green antifouling membranes can be prepared by VIPS, despite the scarcity of green solvents suitable for nonsolvent-induced phase inversion processes. The formation of green PVDF membranes by the VIPS process using zwitterionic copolymers is also under investigation by our team, considering the outstanding antifouling performances of these materials.

## Data Availability

Not applicable.
